# A rare case of a toddler with unilateral cryptorchidism‐related cranial suspensory ligament remnant

**DOI:** 10.1002/ccr3.7310

**Published:** 2023-05-18

**Authors:** Hideaki Nakajima, Kazuto Suda, Atsushi Arakawa, Toshihiro Yanai

**Affiliations:** ^1^ Department of Pediatric Surgery Ibaraki Children's Hospital Mito City, Ibaraki Japan; ^2^ Department of Human Pathology Juntendo University School of Medicine Tokyo Japan; ^3^ Department of Pediatric Urology Ibaraki Children's Hospital Mito City, Ibaraki Japan

**Keywords:** androgen receptor, cranial suspensory ligament, cryptorchidism, gubernaculum, testicular descent

## Abstract

**Key clinical message:**

Several animal experiment studies have shown that insufficient testicular descent to the scrotum can be caused by persistence of cranial suspensory ligament (CSL). We report a case of right cryptorchidism in a male toddler surgically treated with an orchidopexy possibly associated with CSL persistence based on intraoperative and pathological findings. This case would be a precious source to further investigate the etiopathogenesis of cryptorchidism.

**Abstract:**

The CSL anchors embryonic gonads to the dorsal abdominal wall during antenatal mammalian development. Although its persistence appears to cause cryptorchidism in animal models, it has never been proven in humans. A 1‐year‐old boy with right cryptorchidism underwent right orchidopexy. Intraoperatively, a band‐like structure running from the right testis into the retroperitoneum and up to the right side of the liver was noticed and resected. The pathological findings of the specimen showed fibrous connective tissues, smooth muscles, and blood vessels but no tissues suggestive of a testis, a spermatic cord, an epididymis, or liver. Immunohistochemical analysis for an androgen receptor antibody did not detect any signal in the specimen. The right cryptorchidism in this case was possibly caused by CSL persistence, which is the first such human case, to our knowledge.

## Graphical abstract

A band‐like structure is attached to the superior pole of the testis.

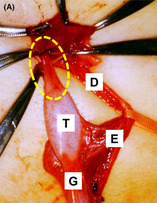

## 1 INTRODUCTION

Testicular descent is known to be preceded by cranial suspensory ligament (CSL) regression and gubernacular development.^1^, ^2^, ^3^ This mechanism facilitates testicular fixation in the scrotum from the antenatal period to several months after birth.^3^ Disorders in these processes can cause cryptorchidism, as proven by experimental studies using animal models.^1^, ^2^ CSL regression in males depends on interaction with signals between androgen and its receptor.^1^ However, no human case of this clinical status has been reported. We report a case of right cryptorchidism in a male toddler surgically treated with a right orchidopexy thought to be associated with CSL persistence based on intraoperative and pathological findings.

## 2 CASE PRESENTATION

A right cryptorchidism, a right inguinal hernia, and a left‐sided retracted testicle were diagnosed in a male toddler. The history of his fetal development and delivery were unremarkable. He developed right inguinal hernia incarceration that required manual reduction at 18 months of age. The family medical history was unremarkable. The physical findings revealed a palpable normal‐sized testis within the right inguinal canal.

**FIGURE 1:**
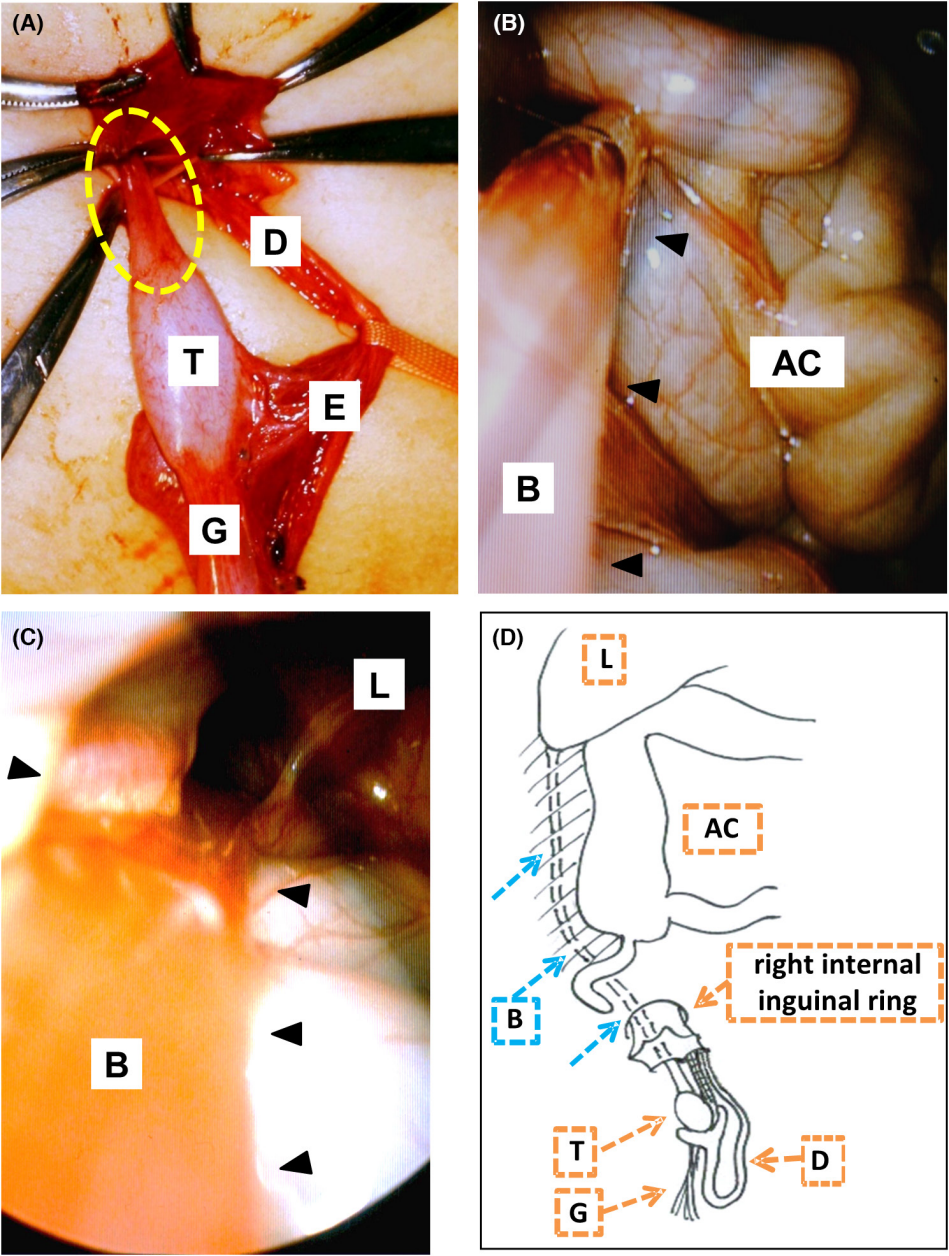
(A) Intraoperative view of the inguinal surgical site. A band‐like structure (dotted ellipse) is attached to the superior pole of the testis. (B, C) Laparoscopic findings are shown. The band is running along the right paracolic gutter (black arrowheads) (B) retroperitoneally up to the retroperitoneum adjacent to the right lobe of the liver (black arrowheads) (C). (D) Scheme showing the operative findings that show the anatomical location of the band (B; blue dotted arrows) and its environment. AC, ascending colon; B, band; D, deferent duct; E, epididymis; G, gubernaculum; L, liver; T, testis.

He was scheduled for routine bilateral orchiopexies at the age of 19 months. For the right side operation, a typical right inguinal incision was made, and a normal‐sized testis was identified in the right inguinal canal. The deferent duct was connected to the testis. However, an epididymal head was not attached to the right testis upper pole (Figure 1A). The gubernaculum attached to the scrotum neck was resected, and detachment of the hernial sac and testis was completed. Following this, a 3‐mm diameter band‐like structure attached to the superior pole of the testis was found (Figure 1A) and ran into the abdominal cavity. Additional laparoscopic observation through the right internal inguinal ring revealed that the band‐like structure ran along the right paracolic gutter retroperitoneally (Figure 1B) up to the retroperitoneum adjacent to the right lobe of the liver (Figure 1C). The band‐like structure was resected near the superior pole of the testis (Figure 1D), and a specimen was sent for histopathological examination. The right inguinal hernia was repaired by high ligation and right orchiopexy after a scrotal incision. Left orchidopexy with another scrotal incision was performed. No abnormal structure was observed directly or laparoscopically on the left side. The postoperative course was uneventful. Both testes were in the scrotum and normal‐sized after 1‐year follow‐up.

**FIGURE 2:**
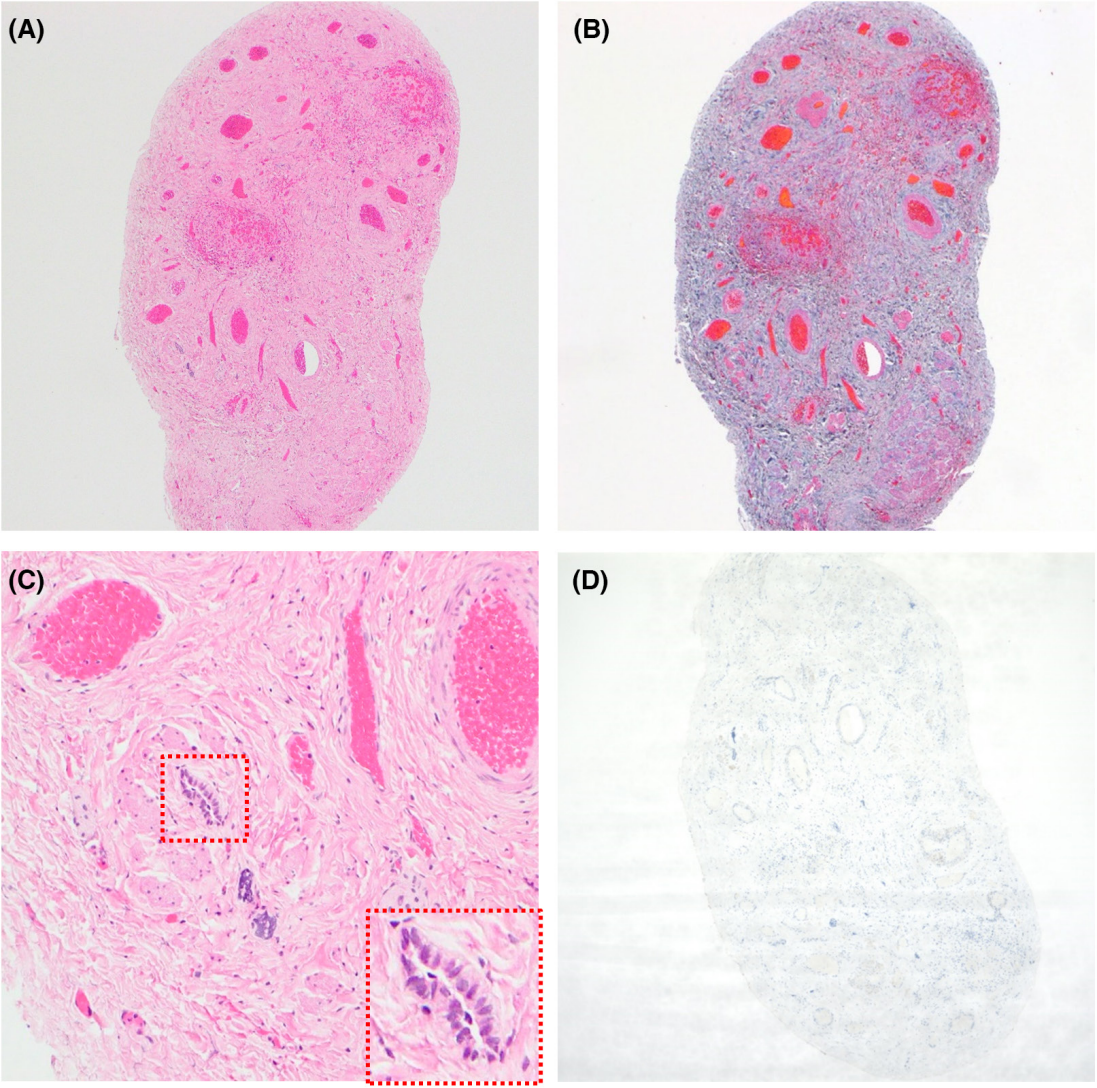
(A) ×40 views of the resected band assessed by hematoxylin and eosin (HE). A lot of fibrous connective tissues and smooth muscle fibers with blood vessels are seen. (B) Masson trichrome (MT) staining of the resected band. The tissue is composed of diffuse fibrous connective tissues and smooth muscles, ×40. (C) ×400 views of the resected band assessed by HE. Luminal structures lined by epithelial cells are seen in the connective tissues (dotted square). (D) Anti‐androgen receptor antibody staining. Signals are not detected from the specimen, ×40.

Hematoxylin and eosin (HE) staining and Masson trichrome staining revealed that the band‐like structure comprised fibrous connective tissues and smooth muscles (Figures 2A,B) containing some luminal structures lined with epithelial cells (Figure 2C) and blood vessels. There were no tissues suggestive of a testis, a spermatic cord, or an epididymis. Immunohistochemical analysis for an androgen receptor antibody (Abcam, ab9474) did not detect any signal in the specimen (Figure 2D). No component indicating liver tissue was detected by HE staining.

## 3 DISCUSSION

The surgical treatment and perioperative course were typical for a patient with cryptorchidism for our patient. Clinically, however, the right cryptorchidism might be implicated with a persistence of CSL remnant according to the band‐like structure between the right testis upper pole and retroperitoneum adjacent to the liver containing fibrous connective tissues and smooth muscles. All of these findings are consistent with previous reports in the literature.^1^, ^4^ CSL is a fibromuscular structure that anchors the embryonic gonad to the dorsal abdominal wall during antenatal mammalian development, including in humans.^1^ Normal testicular descent is facilitated by CSL regression and swelling and shortening of the gubernaculum in the early phase.^2^ Several hormonal regulators are involved with these complex anatomical rearrangements in males such as the androgen–androgen receptor axis causing degeneration of the CSL,^1^ insulin‐like hormone,^2^, ^3^ Müllerian‐inhibiting substance, and anti‐Müllerian hormone causing swelling and shortening of the gubernaculum.^2^ Additionally, outgrowth of the gubernaculum and attachment of the testis in the scrotum occur in the late phase controlled by androgens and calcitonin gene‐related peptides.^2^, ^3^ Despite little information on the role of CSL in testicular maldescent, animal experimental studies have observed cryptorchidism with persistent CSL.^1^, ^4^ One study reported that inhibition of androgen signaling to the CSL and expression of androgen receptor in male rats during the early fetal period resulted in CSL persistence, developing intra‐abdominal or inguinal testes in approximately half of the cohorts.^1^ This likely explains the etiology of cryptorchidism.^1^ These previous findings have similarities to those in our case because we also observed a lack of androgen receptor signaling in the CSL remnant, which might have been caused by maldistribution of androgen receptor expression on the bilateral CSL during tissue development, leading to unilateral cryptorchidism. On the contrary, bilateral cryptorchidism theoretically could develop from systemically insufficient androgen secretion during the fetal period.

These patients might present different types of band‐like structures rather than CSL remnants. For example, a hepatogonadal cord‐like structure characterized by a fetal mesonephric sheath with abnormal attachment between the upper pole of the testis and the inferior surface of the liver could be considered.^5^ The cord arises in the retroperitoneal space and could inhibit testicular descent if the hepatogonadal cord‐like structure was persistent. Furthermore, hepatic tissue, including hepatocytes, can be present along the entire length of its structure according to a previous report.^5^ However, it was unlikely in our case because we found no histological evidence for liver tissue.

## 4 CONCLUSION

According to the anatomical features and histopathological results, we suspected that our patient's condition was associated with a CSL remnant. The findings in this case should be helpful to further investigate the etiopathogenesis of cryptorchidism.

## AUTHOR CONTRIBUTIONS

**Hideaki Nakajima:** Data curation; investigation; project administration; resources; writing – original draft; writing – review and editing. **Kazuto Suda:** Data curation; investigation; resources; writing – review and editing. **Atsushi Arakawa:** Data curation; investigation; resources. **Toshihiro Yanai:** Conceptualization; project administration; supervision; writing – review and editing.

## FUNDING INFORMATION

This research did not receive any specific grant from funding agencies in the public, commercial, or not‐for‐profit sectors.

## CONFLICT OF INTEREST STATEMENT

The authors declare no conflict of interest.

## CONSENT

Written informed consent was obtained from the patient's guardian to publish this report in accordance with the journal's patient consent policy.

## Data Availability

The data that support the findings of this study are available from the corresponding author upon reasonable request.
